# Bringing AI to Clinicians: Simplifying Pleural Effusion Cytology Diagnosis with User-Friendly Models

**DOI:** 10.3390/diagnostics15101240

**Published:** 2025-05-14

**Authors:** Enrico Giarnieri, Elisabetta Carico, Stefania Scarpino, Alberto Ricci, Pierdonato Bruno, Simone Scardapane, Daniele Giansanti

**Affiliations:** 1 Cytopathology Unit, Department of Clinical and Molecular Medicine, Sant’Andrea University Hospital, Sapienza University of Rome, Via di Grottarossa 1035, 00189 Rome, Italy; elisabetta.carico@uniroma1.it; 2 Morphologic and Molecular Pathology Unit, Department of Clinical and Molecular Medicine, Sant’ Andrea University Hospital, Sapienza University of Rome, Via di Grottarossa 1035, 00189 Rome, Italy; stefania.scarpino@uniroma1.it; 3 Respiratory Disease Unit, Sant’Andrea University Hospital, Sapienza University of Rome, Via di Grottarossa 1035, 00189 Rome, Italy; alberto.ricci@uniroma1.it (A.R.); pierdo.bruno@gmail.com (P.B.); 4 Department of Information Engineering, Electronics and Telecommunications, Sapienza University of Rome, Via Eudossiana 18, 00196 Rome, Italy; simone.scardapane@uniroma1.it; 5 Centre Tisp, ISS, 00161 Rome, Italy

**Keywords:** cytopathology, cytology, pleural effusion, lung adenocarcinoma, machine learning, YOLOv8, YOLOv11, CNN, prediction, precision medicine

## Abstract

**Background:** Malignant pleural effusions (MPEs) are common in advanced lung cancer patients. Cytological examination of pleural fluid is essential for identifying cell types but presents diagnostic challenges, particularly when reactive mesothelial cells mimic neoplastic cells. AI-powered diagnostic systems have emerged as valuable tools in digital cytopathology. This study explores the applicability of machine-learning (ML) models and highlights the importance of accessible tools for clinicians, enabling them to develop AI solutions and make advanced diagnostic tools available even in resource-limited settings. The focus is on differentiating normal/reactive cells from neoplastic cells in pleural effusions linked to lung adenocarcinoma. **Methods:** A dataset from the Cytopathology Unit at the Sant’Andrea University Hospital comprising 969 raw images, annotated with 3130 single mesothelial cells and 3260 adenocarcinoma cells, was categorized into two classes based on morphological features. Object-detection models were developed using YOLOv8 and the latest YOLOv11 instance segmentation models. **Results:** The models achieved an Intersection over Union (IoU) score of 0.72, demonstrating robust performance in class prediction for both categories, with YOLOv11 showing performance improvements over YOLOv8 in different metrics. **Conclusions:** The application of machine learning in cytopathology offers clinicians valuable support in differential diagnosis while also expanding their ability to engage with AI tools and methodologies. The diagnosis of MPEs is marked by substantial morphological and technical variability, underscoring the need for high-quality datasets and advanced deep-learning models. These technologies have the potential to enhance data interpretation and support more effective clinical treatment strategies in the era of precision medicine.

## 1. Introduction

### 1.1. Challenges in Diagnostic Pleural Effusion Cytopathology

Malignant pleural effusion (MPE) refers to a paramalignant condition characterized by the presence of neoplastic cells in the pleural fluid. MPE is often associated with the advanced stages of cancers such as lung, breast, and lymphoma [1,2]. Notably, metastasis to the parietal pleura is common in lung adenocarcinoma, which accounts for 45–65% of all lung cancer cases [3]. The onset of pleural effusion in these advanced stages is typically linked to a poor prognosis, with a median survival time (MST) of four months or less. Therefore, early and accurate detection of MPE is essential for determining the most effective treatment strategies.

*Therapeutic Strategies and Prognosis*

The primary treatment for MPE involves thoracentesis, a procedure used to remove pleural fluid and relieve symptoms. However, thoracentesis does not provide a long-term solution, and its effectiveness can be limited. While some patients may not experience recurrence, others face rapid relapse, sometimes within a month of the procedure [4,5,6,7,8,9]. Recurrence signals the need for more aggressive treatment options. Hence, a swift and accurate identification of the origin of pleural effusion is crucial to customize individualized treatment plans, including interventions to manage fluid production and improve symptom control.

*Diagnostic Obstacles and Methods*

Diagnosing MPE typically begins with laboratory tests, including pleural fluid cytology. Although this traditional method remains crucial for distinguishing malignant cells from reactive mesothelial cells, which are commonly seen in non-neoplastic effusions, it can be challenging when the origin of the primary tumor is unclear. The expertise of pathologists plays a key role in increasing the reliability of this diagnostic method. Additionally, collaboration with clinicians provides essential context to help interpret the cytology more accurately [10].

**Figure 1:**
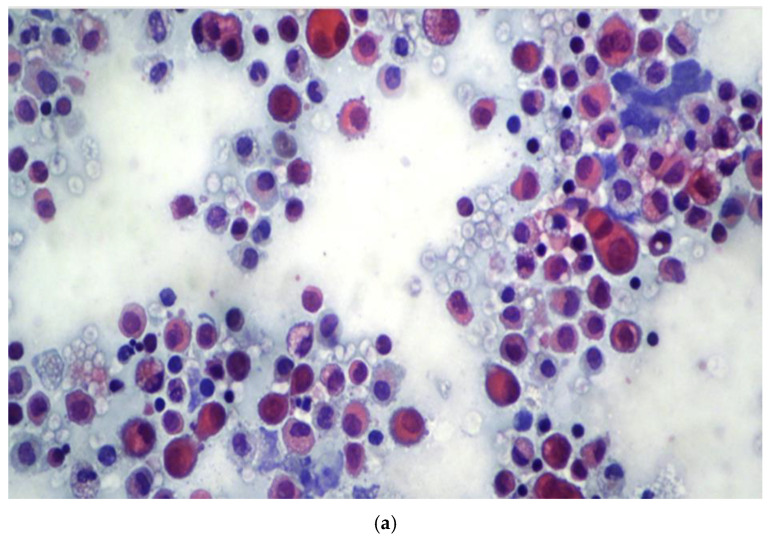

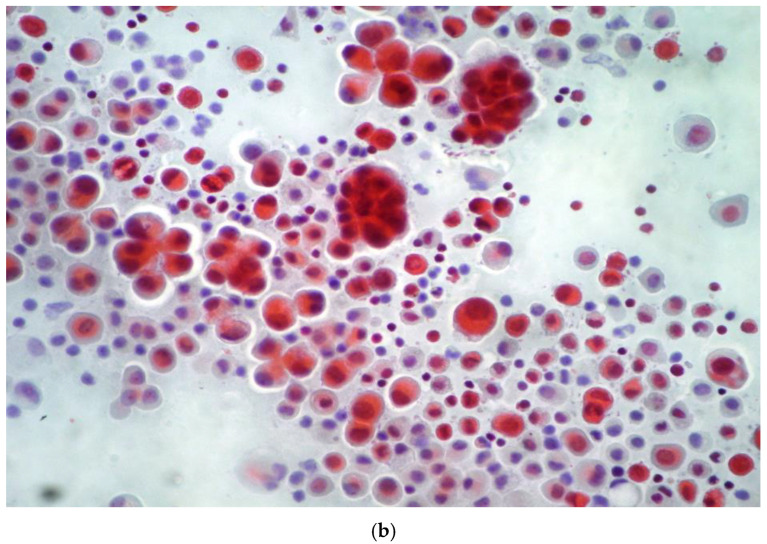
(**a**) Reactive pleural effusion showing mesothelial cells exhibiting degenerative and reactive features (Papanicolaou staining, ×10). (**b**) Adenocarcinoma pleural effusion showing coexistence of mesothelial and neoplastic cells organized in single and cluster aggregates (Papanicolaou staining, ×10). Neoplastic clusters are easily recognizable, whereas single neoplastic cells may resemble mesothelial cells.

However, cytopathology of pleural effusion can be prone to errors, including false positives and false negatives. These inaccuracies are often due to the complex morphology of cells in effusion fluid, which can resemble malignant cells. Reactive changes in mesothelial cells, such as hyperchromatic nuclei, cytoplasmic vacuolization, binucleation, multinucleation, phagocytic activity, and cell-in-cell configurations, can mimic the appearance of malignant cells, particularly those found in adenocarcinoma or mesothelioma. These morphological similarities pose a risk for false-positive diagnoses (Figure 1). This challenge highlights the need for supplementary diagnostic methods to complement traditional morphological analysis.

*The Growing Role of Artificial Intelligence*

In recent years, artificial intelligence (AI) has garnered attention for its potential to improve diagnostic accuracy in cytopathology. Machine-learning (ML) and deep-learning (DL) algorithms, particularly Convolutional Neural Networks (CNNs), have shown promise in analyzing medical images. 

Machine learning is transforming healthcare [11] by enhancing diagnostic accuracy and treatment personalization. It enables the analysis of vast medical datasets to uncover patterns and insights beyond human capability.

By supporting clinical decision-making, it helps reduce errors and improve patient outcomes. ML-driven tools can detect diseases earlier and suggest the most effective interventions. Predictive models assist in identifying at-risk individuals before symptoms appear. Automation through ML also streamlines workflows, saving time and resources.

It facilitates real-time monitoring and adaptive care, especially in chronic disease management. Ultimately, machine learning contributes to a more proactive, precise, and patient-centered healthcare system.

AI models have demonstrated diagnostic accuracy comparable to, or even surpassing, that of human experts [12,13,14]. Initially, these AI models were applied to gynecological cytology, such as Pap smear tests, yielding promising results. However, AI applications have expanded beyond gynecological cytology to include non-gynecological samples, such as those from the urinary tract, breast, thyroid, lung, and pleural metastatic cytopathology [15,16,17,18,19,20,21]. In parallel, the literature is exploring the genetic basis of reproductive health and fertility issues [22]. These studies aim to identify and characterize genes that predispose individuals to both syndromic and non-syndromic forms of infertility, thereby contributing to a better understanding of the molecular mechanisms underlying reproductive disorders. Such insights are not only valuable for diagnostic purposes but also for the development of targeted therapeutic strategies and personalized medicine approaches in reproductive care. Separately, despite the growing application of AI in various areas of cytopathology, research focused specifically on pleural effusion cytology remains limited.

A few studies have applied AI models to distinguish between normal and malignant cells in pleural effusions. These studies have focused on cells from various tumor types, including lung, gastric, and breast adenocarcinoma, using different deep-learning architectures. The results suggest that deep-learning models could enhance the accuracy and efficiency of MPE diagnosis, offering new possibilities for clinical practice.

*Overview of Literature and AI in Pleural Effusion Diagnosis*

Table 1 summarizes the literature on deep-learning-based models aimed at differentiating neoplastic from non-neoplastic pleural effusions in cytopathology. These studies demonstrate the use of AI techniques, such as CNNs, to improve diagnostic accuracy, particularly in distinguishing malignant from benign cells. The success of these studies highlights AI’s potential to assist pathologists in diagnosing MPE more accurately and efficiently [18,21,23,24,25,26,27,28,29,30,31].

### 1.2. Artificial Intelligence in Cytopathology: Enhancing Diagnostic Accuracy Through Cell and Nuclei Segmentation

As cytopathological diagnostic methods continue to advance, particularly with the integration of artificial intelligence (AI), cell and nuclei segmentation in microscopic images has become a critical component of the analysis process. This involves essential tasks like annotating, counting, and tracking cells, which are fundamental for precise diagnoses, especially in the case of pleural effusions. While earlier sections addressed the challenges of identifying malignant pleural effusions, recent developments in AI-driven image analysis are now facilitating more accurate and automated segmentation of cells in pleural fluid samples, further improving diagnostic reliability.

Numerous studies have introduced methods for cell segmentation, which can generally be divided into two main categories: semantic segmentation and instance segmentation. Semantic segmentation involves assigning image pixels to specific objects or regions of interest, such as cells or nuclei, effectively separating these elements from the surrounding background. Some prominent deep-learning models used for semantic segmentation include Fully Convolutional Networks (FCN) and U-Net, which have proven successful in segmenting cell structures in microscopic images [32,33,34].

Instance segmentation, on the other hand, takes segmentation a step further by distinguishing between individual objects within the same class, such as differentiating between multiple cells or nuclei even within the same image. This method generates bounding boxes and corresponding masks, enabling both detection and segmentation in one step. A well-known instance segmentation model is Mask-RCNN, developed by Facebook AI Research (FAIR), which has become one of the leading algorithms for segmenting objects in complex images [35,36]. Various iterations of Mask-RCNN have been created to enhance its performance in cell and nuclei segmentation in biological imaging [37,38,39].

More recently, a transformer-based model called Segment Anything (SAM) has shown outstanding success in image segmentation tasks, setting new performance standards across numerous applications, including cell imaging [40]. SAM’s effectiveness has led to the development of a hybrid approach, CellsSAM, which combines SAM with CellFinder, another transformer-based object-detection model. This hybrid method has yielded improved results for cell image segmentation, offering an innovative solution for the automatic analysis of microscopic images with diverse cellular features.

*YOLO: A Real-Time Solution for Microscopic Image Segmentation*

An important development in the field of AI is also driven by You Only Look Once (YOLO), a family of algorithms originally developed for instance segmentation in natural images (https://medium.com/tag/yolo-object-detection, accessed on 5 April 2025; https://pjreddie.com/darknet/yolo/, accessed on 5 April 2025). YOLO is increasingly being used in medical image analysis (https://github.com/sevdaimany/YOLOv8-Medical-Imaging, accessed on 5 April 2025), due to its speed and accuracy. One of YOLO’s key advantages over traditional models like R-CNN is its ability to detect multiple objects in a single pass. This makes it especially useful for real-time applications that require rapid and efficient analysis of large data sets. Unlike multi-stage models, YOLO performs detection in one unified step, making it particularly valuable for real-time decision-making scenarios, such as in surgical procedures or emergency diagnostics.

The speed and accuracy of YOLO’s object-detection capabilities make it a promising application in analysing pleural effusion samples, the focus of this investigation. By quickly segmenting cells and distinguishing between malignant and benign cells, YOLO algorithms can significantly improve the diagnostic workflow, delivering faster and more reliable results to clinicians. Additionally, YOLO’s capacity to handle a variety of image types and complexities ensures its adaptability to different clinical applications, including the segmentation of cells in microscopic imaging.

### 1.3. Advancing Medical Diagnostics with YOLO: AI-Powered Object Detection in Healthcare

YOLO (You Only Look Once) is an object-detection algorithm that divides input images into a grid system to identify and classify objects within the image. Its architecture comprises three main components: (i) the backbone, (ii) the neck, and (iii) the head [41,42].

1. Backbone: The backbone is responsible for feature extraction. It processes the input image and generates feature maps, which are representations of important patterns and objects in the image [41]. Essentially, the backbone functions as the algorithm’s “eyes”, capturing key elements in the visual data.
2. Neck: The neck acts as an intermediary between the backbone and the head. It aggregates the features extracted by the backbone and refines them through networks like the Feature Pyramid Network (FPN). This component is crucial in improving the resolution and accuracy of object detection, especially in cases with complex object structures [42].
3. Head: The head is responsible for generating the final predictions, such as the coordinates of bounding boxes (the rectangular areas that contain detected objects), objectness scores (indicating the likelihood that an area contains an object), and classification scores (identifying the type of object detected) [42].

YOLO was first introduced in 2015 with the release of YOLOv1 [42], and it quickly gained attention for its ability to perform real-time object detection. Over the years, the algorithm has gone through several updates, with each new version offering improved accuracy, speed, and versatility. These updates culminated in the release of YOLOv11 in 2024 by Ultralytics, which represents the latest iteration of the YOLO algorithm [42].

One of the most significant versions is YOLOv8, which comes in five scaled versions to meet different performance needs: YOLOv8n (nano), YOLOv8s (small), YOLOv8m (medium), YOLOv8l (large), and YOLOv8x (extra-large) [43]. These variations support a range of vision tasks, including the following:

- Object detection: identifying and localizing objects within images.
- Segmentation: dividing images into segments for more detailed analysis.
- Pose estimation: determining the orientation and position of objects, especially human figures.
- Tracking: monitoring the movement of objects across frames in video data.
- Classification: categorizing objects into predefined classes (e.g., car, dog, person) [43].

*YOLO in Medical Applications*

One of the key advantages of YOLO is its versatility, making it highly applicable in fields such as healthcare, where image analysis is critical. In the medical domain, YOLO is used to detect and localize abnormalities in diagnostic images. For example, in cytopathology, where detecting abnormal cells is crucial for accurate diagnosis, YOLO’s object-detection capabilities allow for fast and reliable identification of cancerous cells in tissue samples. This capability can significantly improve the speed and accuracy of diagnoses, particularly in settings with large volumes of medical images that need to be analyzed quickly [44,45].

A summary of various YOLO applications in cytopathology can be found in Table 2, showcasing studies that have used different versions of YOLO, including the latest YOLOv8 and YOLOv11 models [29,45,46,47,48,49,50,51,52,53]. YOLOv11 offers further advancements in computer vision. Like YOLOv8, it supports tasks such as object detection, instance segmentation, classification, keypoint detection, and oriented bounding box (OBB) detection. YOLOv11’s improvements in accuracy and processing speed [54] make it particularly useful in applications requiring high precision and fast analysis, such as medical diagnostics. These advancements make YOLOv11 ideal for real-time applications where quick, accurate results are needed, such as in detecting abnormalities in medical images or identifying tumors in radiographs.

*YOLOv11 and the Future of AI in Healthcare*

Despite being relatively new, YOLOv11 has already demonstrated promising applications in medical fields, although the number of studies specifically in cytopathology is still limited [53]. The ongoing development of YOLO’s design, especially with YOLOv11, is paving the way for even more efficient and accurate AI-driven solutions in healthcare. In cytopathology, where the speed and precision of image analysis are crucial, these advancements can have a significant impact, ensuring faster, more accurate diagnoses and ultimately improving patient outcomes.

By enhancing object-detection capabilities and enabling fast processing, YOLO and its subsequent versions, like YOLOv11, are transforming the landscape of AI in medical diagnostics, making it more accessible, efficient, and effective in clinical settings.

### 1.4. Expanding Access to Artificial Intelligence: Empowering Professionals with Accessible AI Tools for Practical Applications

*In every sector where AI is used, there is a growing need to enable broader participation in its development, rather than limiting involvement to passive use of applications. This represents a real and concrete opportunity for progress. Platforms that contribute to this direction are essential. In every sector where AI is applied, there is an increasing need to promote active involvement in its development, rather than limiting professionals to passive use of existing applications.*

One of the major barriers to the adoption of AI, especially in healthcare and diagnostics, is the technical complexity of training and deploying models. Roboflow, for instance, offers an intuitive platform for building and applying computer vision models, making AI more accessible even to users without programming or data science expertise. This capability is particularly valuable in fields like healthcare, where professionals such as doctors, biologists, and lab technicians, who may lack technical backgrounds, can still benefit from AI support in tasks like image detection and classification.

By allowing users to upload, annotate, and train models on visual datasets, the platform dramatically simplifies the implementation process. This reduces development time and effort and allows users to focus on clinical interpretation rather than algorithmic details.

For example, in digital cytology, object-detection models like YOLO (You Only Look Once) trained on this platform can accurately identify malignant cells in tissue samples, supporting faster and more accurate diagnoses. Integrating such tools into daily workflows enhances efficiency and reduces error margins.

### 1.5. Rationale for the Study and Purpose

This study aims to explore the integration of artificial intelligence (AI) into the diagnostic process of pleural effusion, a medical condition where fluid accumulates in the pleural cavity. Specifically, it focuses on the use of advanced object-detection algorithms like YOLO, particularly its latest versions, YOLOv8 and YOLOv11, to enhance diagnostic accuracy and efficiency in analyzing pleural effusion cell samples. The purpose is to investigate how AI can support cytologists in detecting and classifying abnormalities in pleural effusion samples, providing a solution that requires no deep expertise in data science, thus making AI accessible to all cytopathologists.

*AI for Cytopathologists: Making It Accessible*

The main motivation behind this study is to bridge the gap between complex AI technologies and the needs of cytopathologists working with pleural effusion samples. Cytologists, while experts in their field, often lack the specialized knowledge required to use advanced AI models effectively. AI applications such as YOLO are designed to be user-friendly, enabling cytologists to leverage AI without needing in-depth technical knowledge. This allows professionals to enhance diagnostic processes by utilizing AI-powered models for image analysis, improving accuracy and workflow efficiency.

The integration of AI in pleural effusion diagnostics is especially beneficial because it helps cytologists quickly and reliably distinguish between different cell types, such as malignant and benign cells. This support can play a crucial role in reducing human error, expediting diagnoses, and improving patient outcomes. The adoption of AI in pleural effusion analysis ultimately democratizes the use of advanced technology in medical diagnostics, providing a valuable application for all cytologists.

*YOLO: Key Innovations*

YOLO, which stands for "You Only Look Once," is an object-detection algorithm designed to process images quickly and accurately. Over the years, YOLO has been updated, and the most recent versions—YOLOv8 and YOLOv11—offer significant improvements in speed, precision, and recall, making them highly suitable for medical applications such as cytology.

YOLOv11 introduces optimizations that enhance the accuracy and speed of object detection, classification, and segmentation tasks. These capabilities are particularly valuable in cytopathology, where precision is key to correctly identifying cell types and abnormalities in pleural effusion samples. The model can help cytologists automate the process of identifying and classifying malignant cells, thus improving diagnostic accuracy.

In this study, we focus on evaluating the performance of YOLOv8 and YOLOv11 in detecting and classifying cells from pleural effusion samples. The results show that both models perform well, with YOLOv11 slightly outperforming YOLOv8 in terms of precision and mean Average Precision (mAP). This demonstrates that YOLOv11 can be a powerful application in the analysis of pleural effusion samples, with the potential to aid cytologists in differentiating between malignant and benign cells more effectively.

*The Purpose of This Study*

The primary purpose of this study is to assess the effectiveness of YOLO-based AI models in analyzing pleural effusion samples. By evaluating the latest versions of YOLO, particularly YOLOv8 and YOLOv11, this research aims to determine how these models can enhance the diagnostic capabilities of cytopathologists, improving both the accuracy and efficiency of pleural effusion analysis. The study highlights how AI, specifically YOLO, can be used as an application to support cytologists in identifying and classifying cells without requiring specialized expertise in machine learning.

This research contributes to the broader field of AI in medical diagnostics by demonstrating how AI can be applied to the specific context of pleural effusion analysis. It explores the benefits of using AI models to detect subtle differences between malignant and benign cells, potentially reducing diagnostic errors and aiding early detection of serious conditions. 

Overall, the rationale behind this study is to explore the integration of AI in the diagnosis of pleural effusion, focusing on the use of YOLO-based models. These models can help cytologists analyze pleural effusion samples with greater precision, making AI a valuable application for improving diagnostic workflows. The study underscores the potential for AI to support cytologists in detecting abnormalities in pleural effusion samples, ultimately enhancing the accuracy and efficiency of diagnoses, improving patient care, and making AI accessible to all cytopathologists.

## 2. Materials and Methods

This study utilized advanced computer vision techniques, powered by the YOLO family of deep-learning models, to segment and analyze cells in pleural effusion images. The primary objective was to develop a robust method for distinguishing malignant cells from benign cells in pleural effusions, particularly focusing on lung adenocarcinoma and mesothelial cells. 

The YOLO architecture, known for its efficiency in object detection, was particularly suited for this task as it can perform real-time segmentation, providing both speed and precision in detecting cell structures within microscopic images. This method ensures accurate identification and segmentation of cells and nuclei, critical for diagnosing pleural effusion samples in cytopathology. Below, we describe the process of image collection, dataset preparation, and the specific steps taken in the training and evaluation of the models.

### 2.1. Collection of Images, Dataset Preparation, and Morphological Criteria for Cells

This pilot study retrospectively analyzed cases of benign and lung adenocarcinoma pleural effusions collected at Sant’Andrea Hospital in Rome between January 2022 and December 2023. The dataset comprises 78 cases with pleural effusions, split into 40 cases of lung adenocarcinoma pleural effusions and 38 benign pleural effusions. The slides were selected by senior cytopathologists (EG, EC), and diagnoses were based on cytological features. Immunohistochemical testing (including Calretinin, TTF-1, and Napsin A antibodies) was utilized to confirm or rule out malignant pleural effusions of lung adenocarcinoma origin. Clinical follow-up information was excluded, and all slides were anonymized for evaluation. The samples were prepared using standard methods and stained using Papanicolaou staining. A digital camera DP27 (Olympus) mounted on a BX45 microscope (Olympus, Tokyo, Japan) was employed to capture up to 100 images per slide. The magnification was set at ×200, focus was manually adjusted, and the depth of focus was optimized to capture the entire area of each cell. Color normalization was applied post-capture for consistency. Some images were excluded due to issues such as preparation defects, blurry photos, overlapping cells, pseudopapillary structures, excessive cytoplasm, or disorganized cell clusters. Mesothelial cells selected for the study exhibited features such as nuclear enlargement, a high nucleus-to-cytoplasm ratio, prominent nucleoli, and bi/multinucleation. Adenocarcinoma cells appeared as isolated atypical cells with enlarged, round nuclei, binucleation or multinucleation, and dense cytoplasm, occasionally containing vacuolization.

### 2.2. Annotation, Data Augmentation, Model Training, and Metrics Evaluation

**Figure 2:**
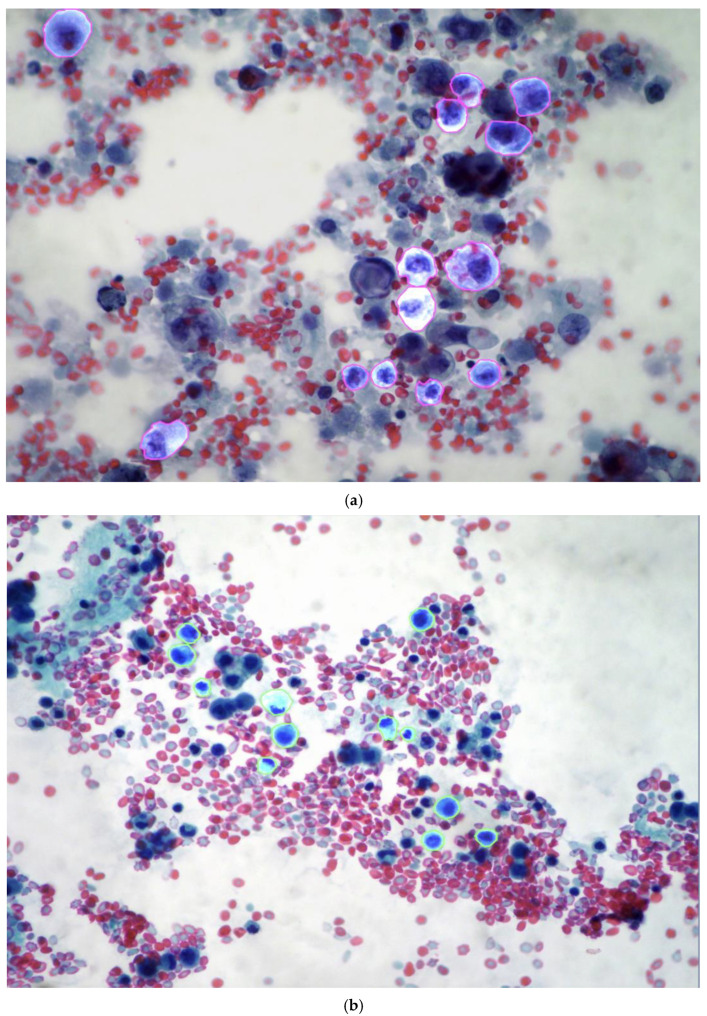
Different cases showing reactive/normal and neoplastic pleural effusion cells were divided into two classes and manually annotated. (**a**) Single adenocarcinoma cells (adk) with violet annotations (Papanicolaou staining). (**b**) Single mesothelial cells (ms) with green annotation (Papanicolaou staining).

The dataset, including image parameters and annotations for each class, is outlined in Table 3. From the collected images, a computer vision dataset was constructed, consisting of 969 manual annotated images showing mesothelial cells (3130) and adenocarcinoma cells (3260) (Figure 2). After annotation, the images were resized to 640 × 640 pixels. 

The dataset was divided into 80% training, 10% validation, and 10% testing subsets, following a commonly adopted convention in biomedical image classification tasks. This split balances the need for sufficient training data with reliable evaluation through separate validation and testing sets. The 80-10-10 ratio has been shown to yield superior performance in several studies. For instance, Golchubian et al. demonstrated that this split achieved higher accuracy (81.6%) compared to a 60-20-20 configuration in a deep-learning model for photo quality classification [55]. Similarly, in cytological image analysis, Boussel et al. employed the same ratio to ensure balanced class distribution across all subsets in the classification of Pap smear cell images [56]. Given the pilot nature of our study and the limited dataset size, the 80-10-10 split was considered both appropriate and methodologically sound to support robust model development and evaluation.

Roboflow technology was used to facilitate the export of the dataset to Google Colab, enabling custom training and evaluation of model performance. Data augmentation techniques were employed to enhance model performance and mitigate overfitting. The images were horizontally and vertically flipped to increase the model’s ability to recognize symmetrical objects. Random cropping allowed the model to identify partially visible objects, and 90-degree rotations were applied to facilitate recognition of cells in various orientations. Following training, performance metrics such as mean Average Precision (mAP) and Precision-Recall (PR) curves were calculated to evaluate the model’s efficacy. mAP, which is used to measure model performance across different thresholds, considers both precision (correctness of predictions) and recall (the model’s ability to identify all relevant instances). Following data augmentation, instance segmentation was performed using YOLOv8 and YOLOv11 models, trained on a Tesla T4 GPU with NVIDIA driver version 535.104.05 and CUDA 12.2.

## 3. Results

In this section, we present the evaluation results of two deep-learning models, YOLOv8 and YOLOv11, applied to the task of classifying pleural effusion cells. The goal of this study is to assess the models’ ability to distinguish between malignant adenocarcinoma cells (adk) and benign mesothelial cells (ms) in pleural fluid samples. The results presented here include a detailed comparison of both models’ performance based on a range of metrics, including precision, recall, F1 score, and mean Average Precision (mAP).

We also examine the models’ performance through a confusion matrix, which provides insight into the classification accuracy for each cell type. Furthermore, we conduct external validation using a public dataset of malignant and benign cells derived from body cavity fluid. This additional validation serves to evaluate the generalization ability of the models when applied to new, unseen data.

The following sections present a breakdown of the quantitative performance of YOLOv8 and YOLOv11, as well as visual examples of the models’ predictions on both the training and external datasets. By evaluating both models’ strengths and weaknesses, we aim to determine which model offers the most reliable classification of pleural effusion cells and explore the potential applications of these models in clinical diagnostics.

### 3.1. Model Training Results: YOLOv8 vs. YOLOv11

The training results from the pleural effusion cell evaluation dataset revealed key performance metrics for both YOLOv8 and YOLOv11, summarized in Table 4. These results highlight the precision, recall, and overall classification performance of both models across two cell classes: mesothelial cells (ms) and adenocarcinoma cells (adk).

The training results obtained from the pleural effusion cell evaluation dataset showed values and metrics categorized by class (Table 4). YOLOv8 demonstrated a precision of 0.673 for all cells, with 0.672 for mesothelial cells (ms) and 0.674 for adenocarcinoma cells (adk), while recall values were 0.784 overall, 0.806 for ms, and 0.761 for adk. The overall performance, measured by the mean Average Precision (mAP), was 0.777. According to the normalized confusion matrix, classification accuracy reached 0.80 for adk and 0.77 for ms, indicating solid model performance in distinguishing between the two cell types.

**Figure 3:**
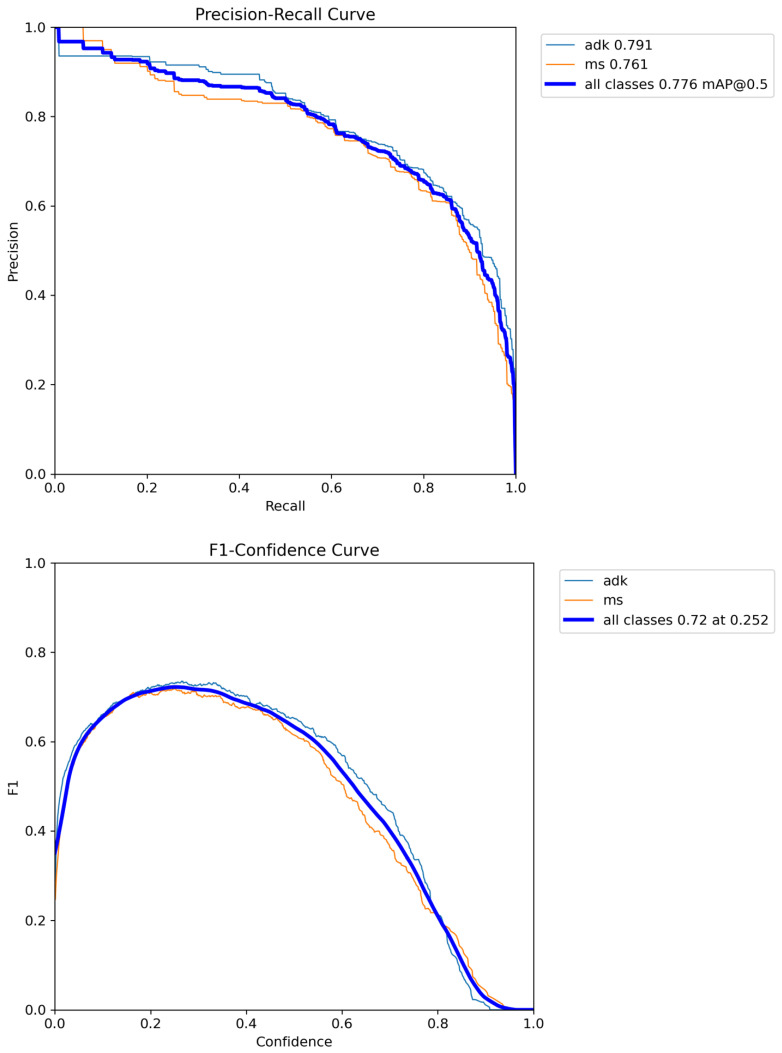

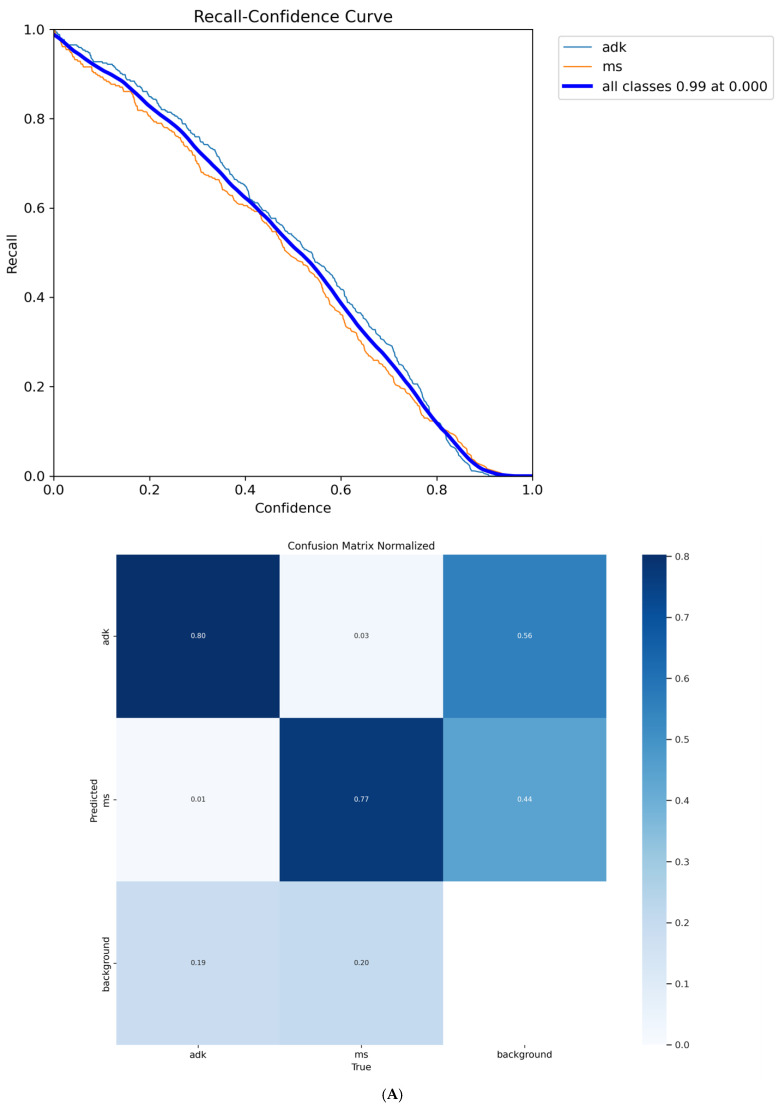

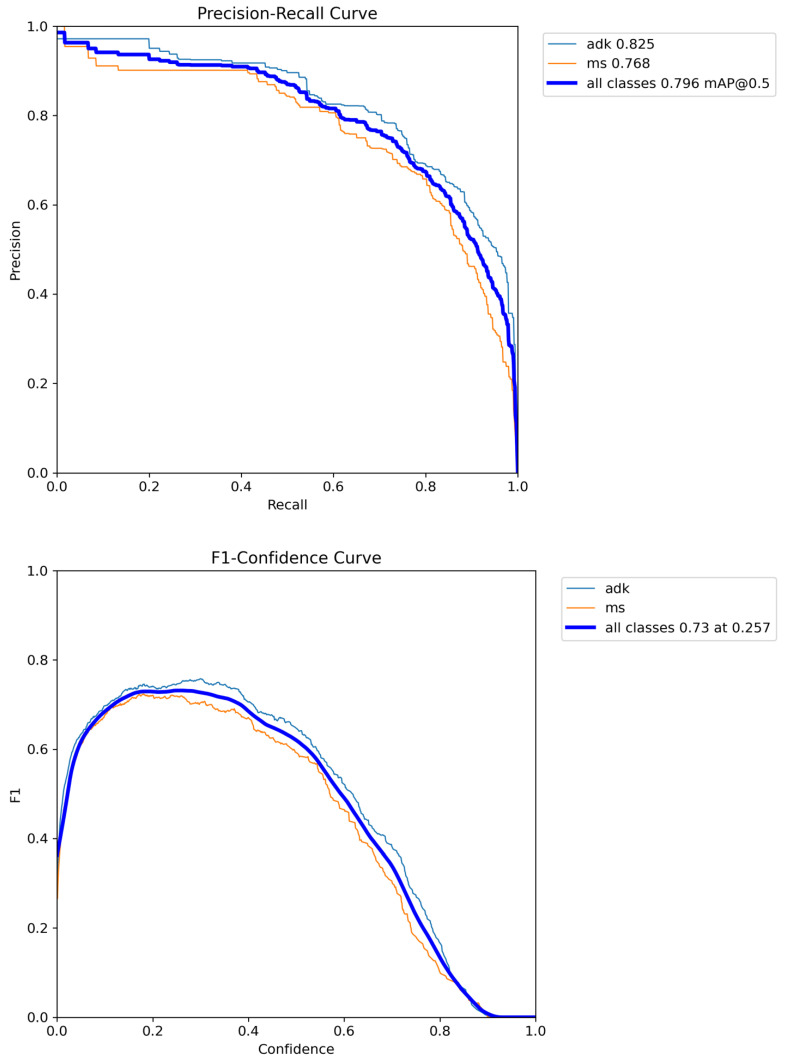

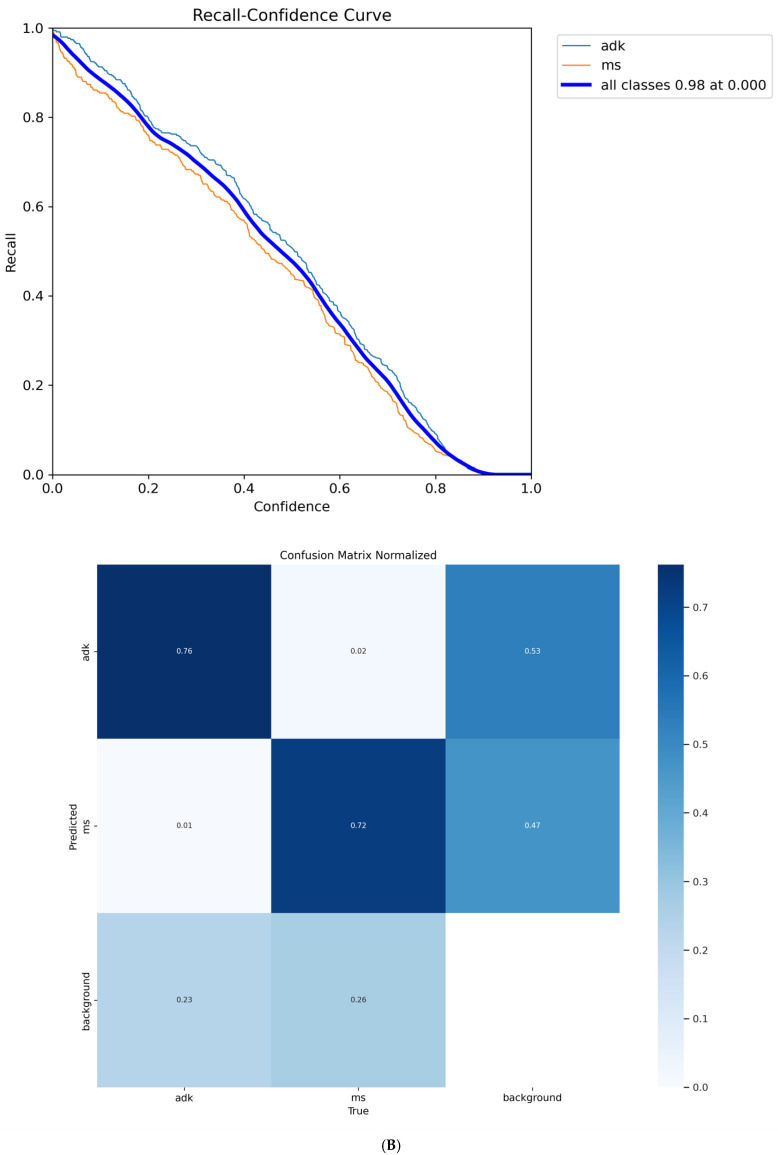
(**A**) YOLOv8 performance metrics. (**B**) YOLOv11 performance metrics.

Compared to YOLOv8, YOLOv11 achieved slightly better results in terms of precision, mAP50, and mAP50-95, reflecting improvements in average classification accuracy across varying thresholds. However, it performed slightly worse in terms of class-specific accuracy, as shown in the confusion matrix (Figure 3A,B), suggesting a possible trade-off between precision and overall class discrimination.

Overall, YOLOv11 emerges as the more favorable model when considering its consistent performance across multiple key metrics. While both models achieved the same F1 score, YOLOv11 demonstrated higher overall precision (0.701 vs. 0.673), indicating a better ability to reduce false positives across classes. Moreover, it outperformed YOLOv8 in both mAP50 (0.796 vs. 0.777) and mAP50-95 (0.681 vs. 0.662), which reflect not only its superior detection accuracy at a standard threshold but also its robustness across varying levels of IoU. These enhancements are especially relevant in clinical contexts, where precision and reliability are essential for supporting diagnostic decisions. Although YOLOv11 showed a slightly lower class-specific accuracy in the confusion matrix, its overall performance profile—particularly in metrics that better capture generalization and model quality—suggests it may be better suited for practical implementation in AI-assisted cytological analysis.

Figure 3A,B provides a visual comparison of the models’ performance metrics. 

Precision-Recall (PR) curves provide a valuable tool to evaluate model performance, especially in datasets with class imbalance, as they focus on the trade-off between precision and recall. This is particularly relevant in medical applications, where detecting true positives (recall) without generating excessive false positives (precision) is critical.

The figures show the performance of the YOLOv8 and YOLOv11 models in classifying mesothelial (MS) and adenocarcinoma (ADK) cells, along with their respective performance curves. The thick, dark blue line indicates the average performance across all classes, while the green and orange lines represent ADK and MS cells, respectively. Both models demonstrated reliable discrimination capabilities, as evidenced by the Precision-Recall (PR) curves that tend to approach the top-right corner of the plot—a typical indicator of a favorable balance between precision and recall, especially in datasets where class distribution and label ambiguity can affect performance.

The F1-confidence curves provide further insights into the interplay between precision and recall across a range of confidence thresholds. These curves help visualize how the models’ predictions behave as the classification threshold changes, particularly in complex scenarios such as those involving overlapping cell boundaries, subtle morphological features, or heterogeneous staining patterns. A decline in the F1 score at specific thresholds can reflect real-world challenges like class imbalance, ambiguous cell features, inconsistent annotations, or the presence of inflammatory or necrotic cells that may confound classification. The presence of such elements in cytological samples is common and underscores the importance of evaluating performance not only at a fixed threshold but across the entire confidence spectrum.

The recall–confidence curves highlight each model’s sensitivity to positive cases, particularly under lower certainty levels. A higher recall at lower thresholds can indicate the model’s capacity to flag potentially relevant detections, which can be beneficial in clinical screening workflows where sensitivity is often prioritized. In this context, one of the models, YOLOv8, shows a slightly higher recall for adenocarcinoma cells compared to the other, suggesting a potential advantage in detecting malignant features, particularly in challenging or ambiguous cases.

Complementing these performance curves, the confusion matrix offers a more granular look at the models’ classification behavior. It includes three categories—adenocarcinoma (ADK), mesothelial (MS), and background—and has been normalized to reflect proportional performance across classes. This normalization accounts for the varying frequencies of each class in the dataset, allowing a fairer assessment of the models’ robustness. The matrix reveals that both models are capable of distinguishing the target cell types with satisfactory accuracy, maintaining low misclassification rates even in challenging conditions. These results support the models’ applicability in real-world cytological analysis, where a balanced trade-off between sensitivity and specificity is crucial.

### 3.2. Evaluation and Validation with External Datasets

To further validate the models’ generalization capabilities, external validation was conducted using a public dataset that contained images of malignant and benign cells derived from body cavity fluid. This validation aimed to test the models’ ability to classify cells accurately when faced with new, unseen data.

**Figure 4:**
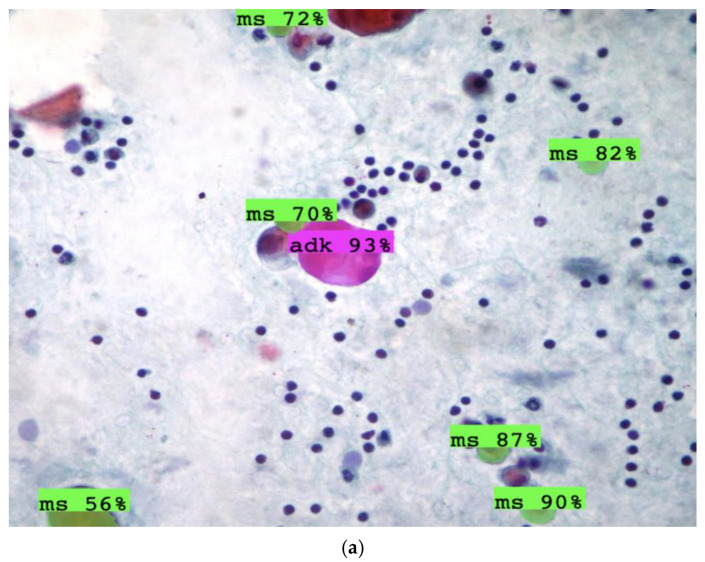

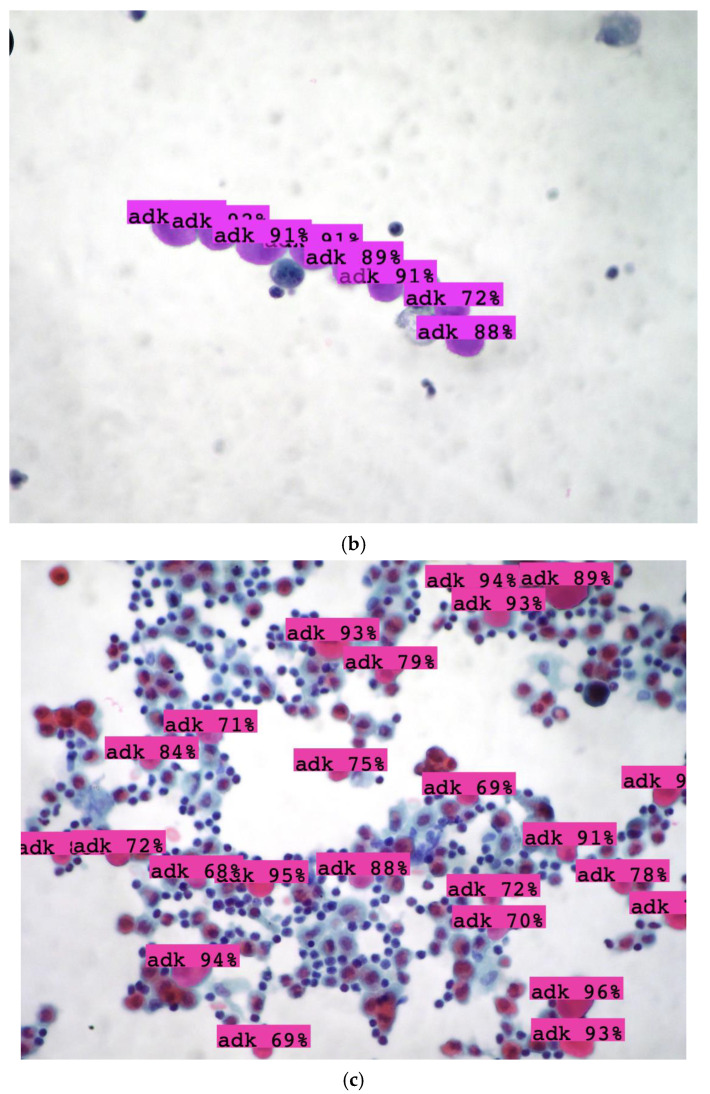
A random selection of images from the test set to verify prediction performance on the two classes of cells at a 50% confidence threshold. (**a**) Isolated adenocarcinoma cells (adk) among mesothelial cells (ms). (**b**) Cluster of adenocarcinoma cells. (**c**) Isolated adenocarcinoma cells in a context of marked inflammation.

A random selection of images from both the training and external datasets was utilized to assess the models’ performance in distinguishing between malignant adenocarcinoma cells (adk) and benign mesothelial cells (ms). These images presented additional challenges, such as overlapping cells and poor image quality, but the models were still able to perform reliably. Figure 4 showcases a random selection of test images used to evaluate the models’ performance at a 50% confidence threshold. The images, which specifically assess the ability to distinguish between adenocarcinoma (adk) and mesothelial (ms) cells, include

- (a) Isolated adenocarcinoma (adk) cells among mesothelial (ms) cells.
- (b) A cluster of adenocarcinoma cells.
- (c) Adenocarcinoma cells within an area of marked inflammation.

These images highlight the challenges that the models face, such as identifying adenocarcinoma cells in inflammatory conditions. However, they also demonstrate that both models are capable of identifying and classifying these cells effectively, even under these complex circumstances.

**Figure 5:**
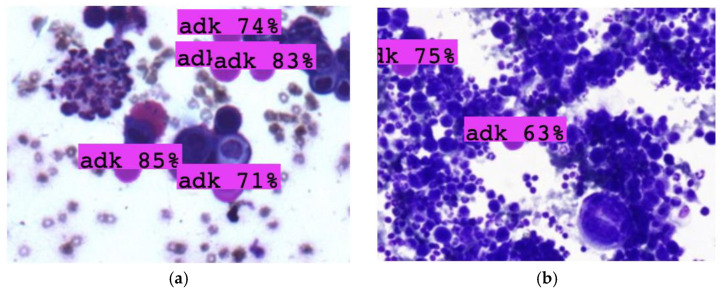

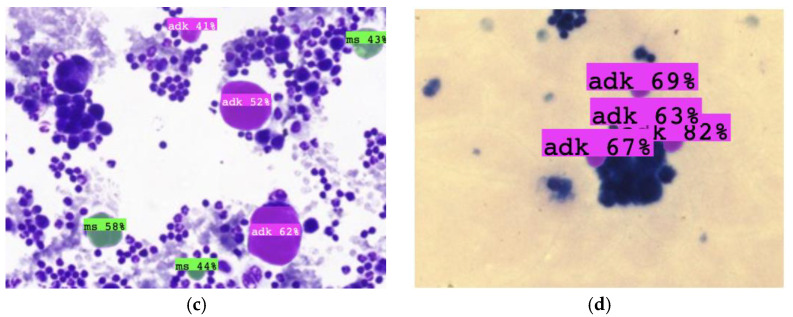
Additional validation was conducted using an external dataset obtained from Kaggle.com containing images of malignant and benign cells derived from body cavity fluid [57]. (**a**–**d**) The results demonstrated predictions consistent with our dataset, even with different staining and magnification. This model could be used in the preliminary observation of and screening to determine whether the sample should be submitted for immunohistochemical evaluation.

Figure 5 presents results from the external dataset, which includes malignant and benign cells derived from body cavity fluid. The external validation confirmed that both YOLOv8 and YOLOv11 were able to accurately classify the cells, even when faced with new images not included in the training dataset. The validation results include

- (a) to (d): Images from the external dataset that were correctly classified by both models, demonstrating consistency with the training dataset.

These results underscore the models’ ability to generalize to new data, which is essential for their deployment in clinical settings. The accurate classification of cells, even when they are not clearly visible or appear in challenging conditions, suggests that the models could be valuable applications for preliminary screening of pleural effusion samples before submitting them for more detailed immunohistochemical evaluation.

### 3.3. Final Performance Summary

In summary, the comparison between YOLOv8 and YOLOv11 reveals that while YOLOv11 marginally outperformed YOLOv8 in precision, mAP, both models demonstrated strong performance in classifying pleural effusion cells. External validation using both test images and an external dataset further supports the models’ ability to generalize to new, unseen data. Figure 3A,B, Figure 4 and Figure 5 provide visual evidence of the models’ robust performance in various real-world scenarios, indicating their potential use in clinical practice for early detection and screening of pleural effusion samples. While both YOLOv8 and YOLOv11 achieved the same F1 score (0.72), a closer analysis of the individual metrics reveals nuanced differences in their performance. YOLOv11 demonstrated higher precision (0.701 vs. 0.673) and superior mean Average Precision values, both at the standard IoU threshold of 50% (mAP50: 0.796 vs. 0.777) and across the full IoU range from 50% to 95% (mAP50-95: 0.681 vs. 0.662). These results indicate that YOLOv11 provides slightly more accurate and consistent predictions overall. Conversely, YOLOv8 achieved a marginally higher recall (0.784 vs. 0.756), suggesting it may be more sensitive in detecting relevant instances, albeit at the cost of a few more false positives. Overall, YOLOv11 appears to strike a more favorable balance between detection precision and localization accuracy, making it a compelling choice for clinical applications where reducing false positives and ensuring consistent classification are key.

## 4. Discussion

### 4.1. Summary and Added Values

This study investigates the application of YOLOv8 and YOLOv11 object-detection models in pleural effusion cytology, focusing on the challenging task of distinguishing between reactive mesothelial and adenocarcinoma cells. YOLOv11 showed slightly better performance in terms of precision and mAP metrics, likely due to architectural improvements. The feasibility of deploying such models in user-friendly environments, such as web-based applications, was explored. This study provides several contributions to the evolving landscape of AI-assisted cytopathology. 

*First*, it offers a focused comparative assessment of two advanced deep-learning architectures applied to a diagnostically demanding context. The subtle morphological differences between reactive mesothelial cells and adenocarcinoma cells in pleural effusions often pose difficulties even to experienced professionals; this study evaluates how state-of-the-art object-detection models address this challenge through both metric-based and visual assessments.

*Second*, the research emphasizes practical issues around dataset creation and enrichment in cytology. By discussing the application of YOLO-based object-detection models and suggesting inter-laboratory collaborations, it highlights important strategies for enhancing the development and implementation of AI systems in healthcare.

*A third contribution* lies in the discussion around expanding access to AI development. The study highlights how user-oriented applications can support data annotation, model training, and integration processes, particularly in fields such as cytology, where dedicated AI development infrastructure is often lacking.

This opens the door to wider participation in AI model development from healthcare professionals without specific computational backgrounds.

*Finally*, by framing the diagnostic model as a potential decision support application in low-cellularity settings such as malignant pleural effusions, the study contributes to the conversation around precision oncology. 

### 4.2. Discussion on the Contribution of the Study to Advancing Diagnostic Techniques in Pleural Effusion

#### 4.2.1. Recent Advances in AI for Pleural Effusion Diagnosis

In recent years, the focus on the diagnostic approach to pleural effusions has shifted, not only within the laboratory but also among clinicians requiring more information beyond the cytomorphological diagnosis. In patients with advanced neoplastic disease, particularly from lung adenocarcinoma, the cells in the effusion represent a valuable resource for determining the most appropriate therapeutic strategies. Tumor cells may be discovered at the initial diagnosis and provide insights into their molecular profile. In some cases, these effusions tend to reaccumulate during or after the course of treatment. In such instances, neoplastic cells become essential, especially for the changes in their genetic profile. Accurate identification and characterization of these cells represent the first challenge even for experienced cytopathologists [58,59,60].

Although artificial intelligence (AI) is mostly used in histopathology, the advancement of deep learning has greatly accelerated the development of computational cytology. In recent years, there has been a booming trend in applying deep learning to screening and classification tasks, localization, identification, and segmentation of different cellular compartments. Most deep-learning-based cytology initially focused on the cervix and later expanded into non-gynecological lesions [61,62,63,64].

In 1995, Truong et al. built an artificial neural network (ANN) model based on densitometric and morphometric data of effusion cells, reporting 95.3% and 85.7% sensitivity and specificity, respectively [65]. In 2012, Barwad et al. applied ANN to effusion cytology using a supervised learning model for network training, identifying both benign and malignant cases [21]. More recently, Xie et al. used a supervised deep-learning method for classifying benign and malignant pleural effusion cells through a deep convolutional neural network (DCNN), achieving accuracy, sensitivity, and specificity of 91.67%, 87.50%, and 94.44%, respectively. They observed that deep-learning methods could assist pathologists, regardless of their experience, in diagnosing cancer cells in pleural effusions [23]. Park et al. applied deep learning to support the diagnosis of metastatic breast carcinoma in pleural effusions, demonstrating that the deep convolutional neural network (DCNN) model outperforms pathologists by showing higher accuracy, sensitivity, and specificity [18].

#### 4.2.2. YOLO Algorithm in Cytopathology

The object-detection algorithm is an important task in computer vision. Traditionally, manual annotation and segmentation of the region of interest (ROI) were time-consuming and error-prone processes. The YOLO (You Only Look Once) algorithm has gained significant attention in the computer vision community. It is a single-stage detector that identifies all objects in an image in a single forward pass through a convolutional neural network (CNN), predicting bounding boxes and class probabilities of objects in an image [66]. YOLO’s application in the medical domain has generated interest due to its ability to enable early diagnosis and detection of various pathologies, including retinal diseases, brain atrophy, abnormal protein deposits, and cardiovascular conditions [67,68,69,70].

Regarding digital pathology, Rong et al. applied YOLO (HD-Yolo) for lung, liver, and breast cancer histology-based detection, accelerating nucleus segmentation and tumor microenvironment quantification [71]. Elazab et al. developed a hybrid model combining the extraction and classification capabilities of ResNet50 with YOLOv5 for glioma tumor diagnosis, demonstrating improved accuracy [72]. Nambu et al. used YOLOv4 in combination with ResNet for detecting atypical cells of the cervix, achieving average accuracy and F-measure values of 90.5% and 70%, respectively [45]. Recently, Ultralytics introduced YOLOv8, a significant advancement for object detection and semantic segmentation, with promising results in various applications. Topuz et al. demonstrated YOLOv7 and YOLOv8’s ability to detect mitotic cells, a critical indicator of cancer aggressiveness [73].

Ikeda et al. used YOLOv8 applied to 11 human cancer cell lines with different staining methods, observing its efficacy in screening and cell classification in clinical settings [74]. YOLOv8 and YOLOv11 have been shown to be highly effective in detecting malignant and benign cells, with recent studies reporting exceptional performance. For example, Awad et al. used YOLOv8 and YOLOv11 to distinguish between malignant and benign white blood cells, achieving an accuracy of 98.8% [53].

#### 4.2.3. Study Contribution and Comparison to the Literature

In our proposed study, we compared the performance of YOLOv8 and YOLOv11 in the diagnostic challenge presented by the strong inter-class similarity between reactive mesothelial and adenocarcinoma cells, particularly when these cells occur singly. The models were validated using both the training dataset and an external dataset, which demonstrated their ability to generalize to unseen data. We also demonstrated the feasibility of integrating these models into a web application for the cell detection process. The metrics results from YOLOv8 and YOLOv11 both showed a good ability to predict the correct class, with YOLOv11 slightly outperforming YOLOv8 in terms of precision, mean Average Precision (mAP50), and mAP50-95 *.

(Note * mAP50 measures the model’s average precision with a minimum overlap threshold of 50%, while mAP50-95 evaluates average precision over a range of overlap thresholds from 50% to 95%, providing a more comprehensive and stringent assessment of model performance. It is important to remember that both average precision (mAP50) and mAP50-95 offer valuable insights into the model’s ability to detect and classify objects, with mAP50 focusing on a specific threshold and mAP50-95 providing a broader evaluation across multiple thresholds). 

The improved performance of YOLOv11 can be attributed to its enhanced architecture, which includes modifications such as better backbone design and more refined feature extraction, making it more effective in distinguishing subtle differences in cell morphology [75].

Additionally, the prediction visualization on individual images derived from a public dataset (Figure 5) showed comparable outcomes. Both YOLOv8 and YOLOv11 achieved accurate cell detection, with YOLOv11 demonstrating a slight edge in robustness, particularly in distinguishing cells with overlapping structures, which is a common issue in pleural effusion samples [76]. Despite the diagnostic challenges and limited dataset, the results obtained are promising. However, several considerations must be made. The drawbacks of the YOLO model include its requirement for a large dataset to train robust AI models and its difficulty in detecting smaller objects, which could lead to potential false positives or false negatives [77]. Furthermore, while YOLOv11 offers a more accurate and reliable prediction, it may still struggle with certain pathological variations that are less frequent or harder to classify due to the complexity of cellular morphology and staining methods used in cytology [78].

Our results align with the growing body of research supporting the integration of deep learning in cytopathology workflows. Notably, previous studies have shown that YOLO-based models can effectively manage the challenges of morphological heterogeneity and overlapping structures in digital cytology, especially in fluid-based preparations such as pleural effusions. For instance, Nambu et al. demonstrated how YOLOv4 combined with ResNet enhanced the detection of atypical cervical cells, reporting substantial gains in accuracy when compared to conventional CNN-based approaches [45]. Similarly, Topuz et al. highlighted YOLOv7’s capacity to identify mitotic figures, a critical feature in cytological assessment of tumor aggressiveness [73]. These studies underscore the suitability of YOLO architectures for tasks that require fine-grained discrimination between cellular subtypes. Moreover, Ikeda et al. applied YOLOv8 to a diverse set of human cancer cell lines and staining conditions, supporting its robustness and transferability across cytological domains [74]. Building on these insights, our study confirms that more recent YOLO iterations, such as YOLOv11, offer concrete advantages in terms of generalization and precision when applied to diagnostically challenging contexts like mesothelial versus adenocarcinoma cell discrimination.

Our findings indicate that YOLOv11 exhibited notable performance improvements over YOLOv8 in metrics particularly relevant to the application of AI models in variable and complex clinical datasets. These enhancements suggest that YOLOv11 may provide a more robust foundation for integrating AI-assisted cytology into routine diagnostic workflows, where consistency, adaptability, and reliable detection are essential.

This aligns with the comparative insights reported by Ultralytics (https://docs.ultralytics.com, accessed on 5 April 2025), supporting the notion that newer YOLO architectures are increasingly refined for deployment in real-world medical contexts.

These findings suggest a potential pathway for the future integration of AI-assisted cytology into clinical settings, where real-time diagnostic support could become a valuable resource, particularly in environments with limited resources or high patient volumes.

However, while larger models such as YOLOv11 and YOLOv8 do require significant computational power, which may not be readily available in many healthcare centers involved in the diagnosis of pleural effusion samples, it is important to note that hospital computational capabilities are advancing rapidly. In recent years, there have been significant improvements in hardware, including the increased availability of GPUs and specialized processing units (e.g., TPUs), as well as the growing use of cloud-based computing solutions. These developments are gradually addressing the challenges related to the high computational demands of deep-learning models.

Moreover, innovations in model optimization, such as quantization and pruning, are making it possible to reduce the size and computational requirements of models like YOLOv11 and YOLOv8, without sacrificing performance. These optimizations could further facilitate the deployment of these models in clinical environments, even in hospitals with more limited computing infrastructure.

As a result, the practical adoption of these models is becoming increasingly feasible, and their potential to enhance diagnostic workflows should not be underestimated. The ability to process large volumes of medical images quickly and accurately can substantially improve the efficiency of diagnoses, enabling healthcare professionals to make more informed decisions in a timely manner. Furthermore, the growing computational capacity within healthcare institutions will likely accelerate the integration of advanced models into routine clinical practice, helping to streamline the diagnostic process and improve accuracy in pleural effusion analysis.

Overall, although computational limitations still represent a challenge, the continuous advancements in hardware and software are progressively mitigating these barriers. As a result, the integration of advanced AI models into clinical practice is becoming increasingly attainable. In particular, such systems have the potential to enhance diagnostic workflows by the following methods:

- Assisting cytopathologists in the preliminary evaluation of cytological samples, effectively acting as a virtual second opinion during the screening phase;
- Reducing the number of cases requiring additional investigations, such as immunohistochemical analyses;
- Lowering overall diagnostic costs and minimizing turnaround times.

### 4.3. Challenges and Future Directions

The increasing availability of user-friendly AI tools is enabling more laboratories and clinical centers to explore deep-learning-based cytology.

This approach not only accelerates AI prototyping but also promotes transparency and data sharing through open repositories. These developments foster the emergence of community-based models and collaborative frameworks that could play a central role in standardizing diagnostic workflows, especially in low-resource or decentralized health networks.

While challenges in collaboration, such as data privacy concerns, geographical barriers, and differences in infrastructure, may arise, these can be addressed through strategic partnerships and innovative solutions. For example, inter-laboratory agreements and consortia can be established to share anonymized data and collectively develop robust datasets. Leveraging cloud platforms and federated learning models can also facilitate the secure and efficient exchange of data across centers, allowing for collaborative efforts without compromising patient privacy.

Looking to the future, Generative Adversarial Networks (GANs) present an exciting opportunity to complement existing methods, particularly in addressing issues like data scarcity and class imbalance. GANs can generate synthetic images that closely resemble real cellular structures, potentially expanding training datasets and improving model performance. For instance, GANs have been used successfully in medical applications, such as generating synthetic histological images [79,80]. In one study, Teramoto et al. employed progressive growing GANs (PGGAN) to classify both real and synthesized lung cytological images, finding that the generated images closely resemble real ones [81]. When integrated with AI models such as YOLO, GANs could enhance the recognition of rare or underrepresented cell types, further improving diagnostic accuracy.

In addition, AI’s integration with molecular biology data offers significant potential for advancing precision medicine. By analyzing tumor cell environments alongside traditional cytological images, machine-learning models could enable more personalized cancer treatment strategies, improving both diagnosis and prognosis, particularly in complex cases like malignant pleural effusions (MPEs), where cell numbers are limited.

As the field progresses, AI is expected to play an increasingly vital role in supporting both diagnostic and therapeutic decisions, providing more accurate, efficient, and personalized approaches to cancer care. Overcoming collaboration barriers through technological solutions and inter-center partnerships will be key to ensuring that AI technologies are implemented effectively and that their benefits are realized across diverse healthcare systems.

### 4.4. Limitation of the Study

This study presents some limitations that should be considered when interpreting the results. The dataset was collected from a single center, the Cytopathology Unit at the Sant’Andrea University Hospital; however, it included a relatively large number of annotated cells for a cytological study, supporting the robustness of the training process. Nonetheless, incorporating multi-center data could enhance morphological diversity and further improve model generalizability.

The analysis focused on cropped and annotated cell images rather than whole-slide images (WSIs). While this approach allowed for accurate object-detection training, future studies could explore how these models perform in real-world diagnostic settings, including WSIs and clinical workflows, where slide quality and imaging variability are important factors. Additionally, although YOLOv11 outperformed YOLOv8 in key metrics, benchmarking against other AI architectures, such as transformer-based or multi-modal models, could offer valuable insights into their comparative diagnostic performance.

Finally, the study did not include explainable AI (XAI) methods. Integrating XAI into future implementations could increase clinical trust and facilitate the adoption of AI-assisted applications in cytopathological practice.

## 5. Conclusions

This study aimed to explore the potential of machine learning, particularly object-detection models, in enhancing the diagnostic process in cytopathology, specifically for distinguishing between mesothelial and adenocarcinoma cells in malignant pleural effusions (MPEs). One of the key objectives was to explore how AI can help make cytopathology more accessible, bringing advanced diagnostic applications within reach of cytopathologists across a wide range of healthcare settings, including those with limited resources. By offering scalable and user-friendly AI solutions, this approach enables clinicians—even those without extensive expertise in data science—to adopt and contribute to the development of AI-based diagnostic tools. This could significantly reduce the technical barriers and empower healthcare professionals worldwide to improve diagnostic efficiency and accuracy, regardless of their technological expertise.

The results demonstrated that both YOLOv8 and YOLOv11 models were effective in detecting mesothelial and adenocarcinoma cells, with YOLOv11 showing slightly better performance in terms of precision and mAP metrics. These findings highlight the potential of machine-learning models to complement human expertise by assisting pathologists in making more accurate and efficient diagnoses. While the final decision will always require human expertise, the integration of machine learning offers significant advantages, such as reducing diagnostic time and potentially enhancing accuracy by revealing subtle morphological differences that may be difficult to detect manually.

Moreover, this study emphasizes the importance of expanding datasets, particularly in fields like cytopathology, where data availability can be a limiting factor. Strategies such as synthetic image generation through Generative Adversarial Networks (GANs) and fostering collaboration between laboratories to share datasets are crucial to overcoming this challenge and improving model robustness. 

Additionally, this study suggests that as future steps, integrating molecular biology data with machine-learning models could further improve diagnostic accuracy. By incorporating biological insights, AI models could enhance the interpretation of cellular features, paving the way for more effective and personalized treatment strategies in the era of precision medicine.

Overall, while AI in cytopathology is still in its early stages, its potential to support diagnostic decision-making, democratize access to advanced applications, and integrate with molecular data offers promising opportunities for improving patient outcomes. The future of AI in healthcare depends on overcoming current challenges, such as dataset limitations and model interpretability, but the progress achieved thus far signals a transformative shift in clinical diagnostics and treatment planning. Looking towards the future, the continued development and integration of AI models in cytopathology offer significant promise for enhancing diagnostic workflows. With platforms empowering clinicians to create and deploy machine-learning models without deep technical expertise, AI has the potential to make advanced diagnostic applications more accessible, even in resource-limited settings. As the field evolves, overcoming challenges such as data limitations and model interpretability will be essential for realizing the full potential of AI in transforming diagnostic practices and improving patient care.

## Figures and Tables

**Table 1:** Deep-learning literature overview in neoplastic and non-neoplastic pleural effusion cytology.

Reference	Pleural Effusion Data Set	Cell Detection Approaches	Metrics
Xie et al. [23]	Lung adenocarcinoma	ResNet-18	AUC 0.67–0.83
Park et al. [18]	Breast cancer	Inception-ResNet-V2	Sensitivity 95% Specificity 98.6% Accuracy 81.1%
Wang et al. [24]	Malignant/nonmalignant cells	ViT vision transformer ResNet-50 Vgg-16 Fundus-DeepNet	Accuracy 96.8 Accuracy 87.3 Accuracy 83.9 Accuracy 88.7
Ren et al. [25]	Lung adenocarcinoma Breast cancer Gastric adenocarcinoma	ResNet-2	AUC 0.807 AUC 0.737 AUC 0.955
Chen et al. [29]	Lung adenocarcinoma	Inception v3 Yolov4	mAP 20% Accuracy 98%
Baykal et al. [30]	Pleural effusion Nuclear detection	Faster R-CNN with ResNet-101	Precision 99.34% Recall 96.93%
Mavropoulos et al. [31]	Breast cancer	Inception v3	AUC 0.96

**Table 2:** Summary of YOLO models application in cytopathology.

Reference	YOLO Model	Cytopathology Application
Chen et al. [29]	YOLOv4	Pleural effusion cytology
Nambu et al. [45]	YOLOv4	Cervical cytology
Wu et al. [46]	YOLOv5	Bronchoalveolar lavage cells
Shi et al. [47]	YOLOv4	Cervical cytology
Tarimo et al. [48]	YOLOv5	Blood cells
Wijaya et al. [49]	YOLOv8	Cervical cytology
Wang et al. [50]	YOLOv7	Lung cancer cytology (biopsy)
Terasaki et al. [51]	YOLOv5	Endometrial cytology
Rumpf et al. [52]	YOLOv7	Bronchoalveolar lavage cells
Awad et al. [53]	YOLOv8-YOLOv11	Blood cells (ALL)

**Table 3:** Dataset analytics and overview of the number of annotations of each class.

**Images** 969	**Annotations** 6390	**Average size** 3.92 mp	**Median image ratio** 2288 × 1712
**All splits** adk cells 3260 ms cells 3130	**Training set** adk 2573 ms 2468	**Validation set** adk 345 ms 309	**Test set** adk 342 ms 353

**Table 4:** Comparison of YOLOv8 and YOLOv11 metrics for all classes.

Models	F1 Score	Precision	Recall	mAP50	mAP50-95
YOLOv8	0.72	0.673	0.784	0.777	0.662
YOLOv11	0.72	0.701	0.756	0.796	0.681

## Data Availability

The datasets of images used to support the findings of this study are available from the corresponding author upon reasonable request.
